# Association of oxaliplatin-containing adjuvant duration with post-treatment fall-related injury and fracture in patients with stage III colon cancer: a population-based retrospective cohort study

**DOI:** 10.1186/s12885-024-12558-2

**Published:** 2024-07-22

**Authors:** Colin Sue-Chue-Lam, Christine Brezden-Masley, Rinku Sutradhar, Amy Ying Xin Yu, Nancy Noel Baxter

**Affiliations:** 1 Division of General Surgery, Department of Surgery, University of Toronto, Toronto, ON Canada; 2 Institute of Health Policy, Management and Evaluation, University of Toronto, Toronto, ON Canada; 3 Division of Medical Oncology, Sinai Health System, Mount Sinai Hospital, Toronto, Canada; 4 ICES, Toronto, ON Canada; 5 Department of Medicine (Neurology), University of Toronto, Sunnybrook Health Sciences Centre, Toronto, ON Canada; 6 Department of Surgery, University of Toronto, Toronto, ON Canada; 7 Li Ka Shing Knowledge Institute, St. Michael’s Hospital, Toronto, ON Canada; 8 Melbourne School of Global and Population Health, University of Melbourne, 207 Bouverie St. Level 5, Melbourne, VIC 3053 Australia

**Keywords:** Colon cancer, Adjuvant therapy, Oxaliplatin, Falls, Neuropathy, Retrospective cohort study

## Abstract

**Purpose:**

Oxaliplatin-containing adjuvant chemotherapy yields a significant survival benefit in stage III colon cancer and is the standard of care. Simultaneously, it causes dose-dependent peripheral neuropathy that may increase the risk of fall-related injury (FRI) such as fracture and laceration. Because these events carry significant morbidity and the global burden of colon cancer is on the rise, we examined the association between treatment with a full versus shortened course of adjuvant chemotherapy and post-treatment FRI and fracture.

**Methods:**

In this overlap propensity score weighted, retrospective cohort study, we included patients aged ≥ 18 years with resected stage III colon cancer diagnosed 2007–2019 and treated with oxaliplatin-containing adjuvant chemotherapy (oxaliplatin plus a fluoropyrimidine; capecitabine [CAPOX] or 5-fluorouracil and leucovorin [FOLFOX]). Propensity score methods facilitate the separation of design from analysis and comparison of baseline characteristics across the weighted groups. Treatment groups were defined as 50% (4 cycles CAPOX/6 cycles FOLFOX) and > 85% (7–8 cycles CAPOX/11–12 cycles FOLFOX) of a maximal course of adjuvant chemotherapy to approximate the treatment durations received in the IDEA collaboration. The main outcomes were time to any FRI and time to fracture. We determined the subdistribution hazard ratios (sHR) estimating the association between FRI/fracture and treatment group, accounting for the competing risk of death.

**Results:**

We included 3,461 patients; 473 (13.7%) received 50% and 2,988 (86.3%) received > 85% of a maximal course of adjuvant therapy. For post-treatment FRI, median follow-up was 4.6 years and total follow-up was 17,968 person-years. There were 508 FRI, 301 fractures, and 692 deaths. Treatment with > 85% of a maximal course of therapy conferred a sHR of 0.84 (95% CI 0.62–1.13) for post-treatment FRI and a sHR of 0.72 (95% CI 0.49–1.06) for post-treatment fracture.

**Conclusion:**

For patients with stage III colon cancer undergoing treatment with oxaliplatin-containing adjuvant chemotherapy, any potential neuropathy associated with longer durations of treatment was not found to result in greater rates of FRI and fracture. Within the limits of this retrospective study, our findings suggest concern about FRI, while mechanistically plausible, ought not to determine treatment duration.

**Supplementary Information:**

The online version contains supplementary material available at 10.1186/s12885-024-12558-2.

## Introduction

While oxaliplatin chemotherapy is recommended by guidelines for the adjuvant treatment of colon cancer, it causes serious, dose-limiting peripheral neuropathy (PN) [1, 2]. The severity of oxaliplatin-associated PN varies from mild numbness or tingling experienced by up to 99% of patients treated with the drug to disabling symptoms that can preclude further treatment and persist for years after treatment [3–6]. Despite the known relationship between PN and gait and balance, few studies have examined the potential relationship between differing durations of oxaliplatin-containing adjuvant chemotherapy and fall-related injury (FRI) – a substantial and potentially avoidable source of morbidity and mortality [7–11].

The potential association between extent of oxaliplatin treatment and risk of post-treatment FRI is relevant for patients and clinicians deciding between 3 versus 6 months of adjuvant therapy for stage III colon cancer after the publication of the International Duration Evaluation of Adjuvant Chemotherapy Collaboration (IDEA) results [12]. An international survey of oncologists found that half reported no greater confidence in decision-making around adjuvant therapy after the publication of IDEA, indicating that in practice this treatment decision remains difficult and may be sensitive to knowledge of additional harms and benefits of differing treatment durations [13]. Moreover, this decision is of lasting importance given that a subset of patients will develop chronic PN, potentially putting them at increased risk for falls into their older years when they are most vulnerable to FRI [5].

The existing literature describing the relationship between neurotoxic chemotherapy and FRI has found an association between FRI and treatment with neurotoxic agents as compared with treatment without any neurotoxic agents [14–18]. However, it is unknown if a relationship exists when comparing durations of chemotherapy in a cohort of patients who all receive neurotoxic chemotherapy. Thus, we have designed a population-based, retrospective cohort study using linked health administrative databases to investigate the association between receipt of 50% versus > 85% of a maximal course of oxaliplatin-containing adjuvant chemotherapy and long-term, post-treatment FRI and fracture in routine practice. These treatment groups were chosen to approximate the treatment durations in the IDEA collaboration.

## Methods

### Study design and population

This retrospective cohort study using routinely collected data is reported in accordance with the RECORD statement and checklist (Online-only Table 1) [19].

Health services and population databases held at ICES (formerly the Institute for Clinical Evaluative Sciences) were used to ascertain covariates, exposures, and outcomes. These datasets were linked using unique encoded identifiers and analyzed at ICES. ICES is an independent non-profit institution with protected legal status allowing it to analyze routinely collected health service and population data.

We included Ontario residents aged ≥ 18 at the time of incident stage III colon adenocarcinoma diagnosis in the Ontario Cancer Registry (OCR) between January 1 2007 and December 31 2019 (Online-only Table 2). The OCR registers incident cancers in Ontario from 1964 onwards and is over 95% complete [20]. The year 2007 was chosen as a cutoff because prior to this date, Ontario staging data are limited and oxaliplatin for adjuvant treatment of colon cancer was not funded by the Ontario Health Insurance Program (OHIP).

Vital status was obtained from the Registered Persons’ Database (RPDB). Individuals were excluded if they had a diagnosis date after their death date, previous cancer diagnosis within 5 years of colon cancer diagnosis, a previous colon cancer diagnosis at any time, multiple simultaneous primary colon cancer diagnoses, non-adenocarcinoma histology, stage IV disease, no curative colon cancer resection within 6 months of diagnosis, previous chemotherapy within 5 years of colon cancer diagnosis, previous oxaliplatin treatment at any time, less than 2 years OHIP coverage prior to the date of first oxaliplatin treatment, or treatment with 0 or > 12 cycles of oxaliplatin. All codes used to define the cohort are listed in Online-only Table 2.

### Exposure

Treatment groups were defined as 50% (4 cycles of CAPOX or 6 cycles of FOLFOX) and > 85% (7–8 cycles of CAPOX or 11–12 cycles of FOLFOX) of a maximal course of oxaliplatin-containing adjuvant chemotherapy to approximate the treatment durations actually received by patients in the IDEA collaboration [21]. Oxaliplatin cycles were ascertained from the Drug Funding Program (NDFP) database, which records all oxaliplatin administered in Ontario. To assign patients to treatment groups, oxaliplatin cycles were captured from the first day of adjuvant treatment through 270 days later. Time zero (index date), the time from which each patient was followed for outcomes, was also set at 270 days after adjuvant initiation to avoid immortal time bias [22]. Based on provincial treatment standards and medical oncologist guidance, this window was sufficient to capture the full course of adjuvant therapy [23].

Patients who stopped oxaliplatin for over 16 weeks and subsequently resumed within the exposure window were excluded since they were likely being treated for recurrence. Patients who died, lost OHIP coverage, received 0 cycles of oxaliplatin, or received > 12 cycles of oxaliplatin during the 270-day exposure window were also excluded from analysis.

### Outcome

The primary outcomes were time from index date to (1) FRI and (2) fracture. FRI was ascertained from NACRS and DAD using the International Classification of Diseases 10th revision (ICD-10) cause of injury codes W00-W19 corresponding to any injury caused by a fall in emergency or inpatient records (e.g. ‘Fall on same level from slipping’) [24]. ICD-10 cause of injury codes always appear with another code corresponding to the type of injury sustained. The positive predictive value of this algorithm for falls is 0.91 (0.86 to 0.94) against a reference standard of chart abstraction [24]. As an alternative measure of FRI, fracture was ascertained from NACRS, DAD, and OHIP using ICD-10, Canadian Classification of Health Interventions (CCI), and OHIP procedure billing codes (Online-only Table 2). For the fracture outcome, all fractures were included irrespective of the presence or absence of cause of injury codes. For each outcome, we were interested in the time to its first occurrence.

### Covariates

We identified potential confounders based on clinical knowledge and existing literature (Online-only Table 2) [25, 26]. Lookback began at the time of the first adjuvant oxaliplatin treatment record and extended back 2 years for frailty, The Johns Hopkins ACG^®^ System Version 10 Aggregated Diagnosis Groups (ADG) comorbidity score, osteoporosis, stroke, alcohol-related hospital visits, pre-treatment neuropathy, and pre-treatment FRI [27]. Age, sex, material deprivation index quintile, rurality, diabetes, and dementia were defined at the time of the first adjuvant oxaliplatin treatment record.

Age (continuous), sex, and rural residence were defined by the RPDB. Using the patient’s most recent address at the time of first oxaliplatin treatment, the Ontario Marginalization Index (ONMARG) defined patients’ dissemination area-level material deprivation index quintile [28]. The index includes measures of education, income, employment, housing quality, and family structure.

Cancer characteristics including the date of diagnosis, cancer site (proximal versus distal), and tumor risk (high-risk T4 or N2 versus low-risk T1-3 and N1) were ascertained from the OCR (Online-only Table 2). Treatment characteristics included time from diagnosis to surgery, time from surgery to adjuvant initiation, 30-day postoperative complications, adjuvant regimen, oxaliplatin dose reduction, and chemotherapy complications (Online-only Table 2).

We defined comorbidity level using ADG comorbidity score (continuous), calculated from weighted ADG categories derived using data from DAD, NACRS, and OHIP [29]. Frail patients were identified using the ACG^®^ System Frailty flag, which defines patients as frail in the presence of diagnostic codes strongly associated with marked functional limitation [30]. Diabetes and dementia were ascertained from validated, ICES derived cohorts contained in the Ontario Diabetes Database and the DEMENTIA database, respectively [31]. Osteoporosis, stroke, and alcohol-related hospital visits were ascertained from OHIP, DAD, and NACRS databases using previously defined coding algorithms [32–36]. To identify pre-treatment FRI, we used the same coding algorithm used to ascertain FRI as an outcome [24]. Pre-treatment neuropathy was defined as at least one visit recorded in NACRS or DAD where the primary diagnostic code corresponded to neuropathy, consistent with a previous study of PN after treatment with oxaliplatin-containing adjuvant chemotherapy (Online-only Table 2) [34]. As the index date was taken to be 270 days after adjuvant treatment initiation, all covariates mentioned above were considered baseline (fixed) measures.

### Statistical analysis

Continuous variables were summarized as means and categorical variables were summarized as proportions for the entire cohort and by exposure group. Standardized differences quantified imbalance between exposure groups. In the overall cohort, we calculated crude event rates per 100 person-years of follow-up and 95% confidence intervals based on lognormal distributions for each outcome.

Overlap weights accounted for confounding by baseline differences between those who received 50% and > 85% of a maximal course of oxaliplatin-containing adjuvant therapy [37]. Propensity scores were first estimated using logistic regression, with treatment assignment as the binary dependent variable and including all patient characteristics as covariates. The estimated propensity score was then used to calculate the overlap weight. Adequacy of the propensity score specification was assessed using propensity score distributions, standardized differences (SD) < 0.10 indicating negligible imbalance, and cumulative distribution functions for continuous variables [38].

The subdistribution hazard ratios (sHR) for the association between treatment with > 85% versus 50% of a maximal course of therapy and each outcome were estimated using overlap-weighted Fine and Gray regression models, with death treated as a competing risk. We chose the competing risk approach over the cause-specific approach as we are most interested in identifying population risk rather than establishing an etiological link [39]. Starting from the index date (270 days after adjuvant initiation), follow-up terminated at the outcome of interest, death, or censoring (loss of OHIP eligibility or end of follow-up [February 28 2022]), whichever came first. Outcomes occurring in the 270 days after adjuvant initiation (prior to the index date) were thus not included in the analysis.

Cumulative incidence functions (CIF), with death as a competing risk, were estimated to illustrate the risk for each outcome over time by exposure group. Robust variance estimators accounted for the overlap weighting procedure [40]. For each outcome, we tested for multiplicative and additive interaction between treatment group and potential effect modifiers of age at diagnosis, sex, and tumor risk [25]. To evaluate multiplicative interaction, we tested an interaction term of the effect modifier and oxaliplatin cycles. Additive interaction was evaluated by determining the *p*-value for the relative excess risk due to interaction (RERI) [41].

The missing indicator approach was used for handling missing deprivation quintile; the proportion missing was extremely small and was assumed to be missing not at random [42]. Complete cases were analyzed for missing rurality and tumor risk, as these were assumed to be missing completely at random.

All statistical tests were two-sided and *p* < 0.05 was considered statistically significant. All analyses were conducted using SAS Enterprise Guide, version 7.1 (SAS Institute Inc., Cary, NC) and R (R Foundation for Statistical Computing, Vienna, Austria).

### Sensitivity analyses

First, we evaluated the association between FRI and treatment with 50% versus 100% of a maximal course of therapy to examine for a dose effect. Second, as FRI may occur repeatedly for each patient throughout their observation window, we examined this outcome as a recurrent event. This was done by fitting overlap-weighted Andersen-Gill recurrent event regression models to estimate the relative rate of FRI [43]. Third, because of the potential for decay in the neurotoxic effect of oxaliplatin to act as a confounder, we included the number of days between the last oxaliplatin cycle and the index date as a fixed covariate in the overlap-weighted Fine and Gray subdistribution hazards regression model for FRI.

## Results

**Fig. 1:**
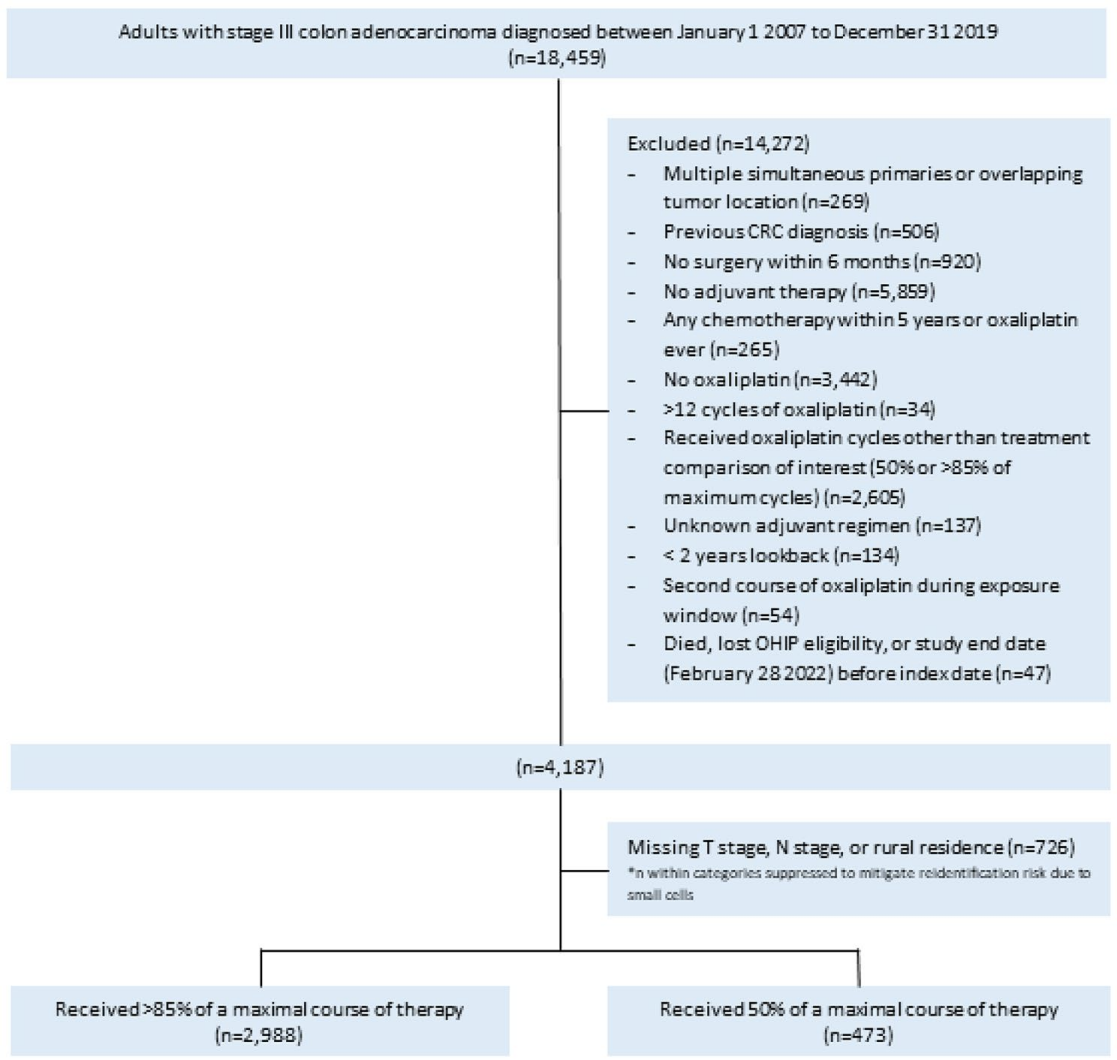
Study flow diagram

After exclusions, 3,461 patients were included in the study, of whom 473 (13.7%) received 50% and 2,988 (86.3%) received > 85% of a maximal course of oxaliplatin-containing adjuvant therapy (Fig. 1). In the unweighted cohort, those who received 50% of a maximal course of therapy were older (mean 61.8 years versus 60.0 years, SD 0.18), less often diagnosed with high-risk tumors (24.9% versus 54.3%, SD 0.63), less likely to experience an oxaliplatin dose reduction (23.0% versus 36.7%, SD 0.30), and more likely to be diagnosed later in the study period (Table 1).

**Table 1:** Distributions of baseline clinical and demographic characteristics of the cohort treated with > 85% or 50% of a maximal course of oxaliplatin-containing adjuvant chemotherapy. In the weighted cohort, patients are weighted by the overlap propensity score weight

Characteristic	All (*N* = 3,461)	Unweighted Cohort, No. (%)			Weighted Cohort, %		
		50% of maximal course (*N* = 473)	> 85% of maximal course (*N* = 2,988)	Std Diff	50% of maximal course	> 85% of maximal course	Std Diff
Age, mean (IQR)	60.3 (54–68)	61.8 (56–69)	60.0 (53–67)	0.18	61.5	61.5	0.00
Male sex	1,961 (56.7)	275 (58.1)	1,686 (56.4)	0.03	57.3	57.3	0.00
ADG score, mean (IQR)	28.7 (22–35)	28.9 (21–35)	28.7 (22–35)	0.02	29.0	29.0	0.00
Frail	111 (3.2)	21 (4.4)	90 (3.0)	0.08	4.4	4.4	0.00
Diabetes	632 (18.3)	82 (17.3)	550 (18.4)	0.03	18.3	18.3	0.00
Dementia	10–14	≤ 5 (0.2-1.0)	9 (0.3)	0.02	0.5	0.5	0.00
Osteoporosis	98 (2.8)	11 (2.3)	87 (2.9)	0.04	1.5	2.5	0.00
Stroke	14–18 (0.4–0.5)	≤ 5 (0.2-1.0)	13 (0.4)	0.04	0.7	0.7	0.00
Alcohol-related hospital visit	22–26 (0.6–0.8)	≤ 5 (0.2-1.0)	21 (0.7)	0.07	0.7	0.7	0.00
Pre-treatment neuropathy	18–22 (0.5–0.6)	≤ 5 (0.2-1.0)	17 (0.6)	0.06	0.3	0.3	0.00
Material deprivation quintile							0.00
1 (least deprived)	753 (21.8)	122 (25.8)	631 (21.1)	0.11	24.3	24.3	0.00
2	681 (19.7)	76 (16.1)	605 (20.2)	0.11	16.4	16.4	0.00
3	701 (20.3)	100 (21.1)	601 (20.1)	0.03	20.5	20.5	0.00
4	712 (20.6)	94 (19.9)	618 (20.7)	0.02	20.4	20.4	0.00
5 (most deprived)	590 (17.0)	77 (16.3)	513 (17.2)	0.02	17.4	17.4	0.00
Missing	24 (0.7)	≤ 5 (0.8)	20 (0.7)	0.02	1.0	1.0	0.00
Rural	459 (13.3)	51 (10.8)	408 (13.7)	0.09	11.3	11.3	0.00
High-risk tumor	1,741 (50.3)	118 (24.9)	1,623 (54.3)	0.63	32.9	32.9	0.00
Proximal tumor (versus distal)	1,781 (51.5)	230 (48.6)	1,551 (51.9)	0.07	48.9	48.9	0.00
Pre-treatment FRI	137 (4.0)	21 (4.4)	116 (3.9)	0.03	4.4	4.4	0.00
Diagnosis year							
2007–2011	987 (28.5)	50 (10.6)	937 (31.4)	0.53	14.3	14.3	0.00
2012–2015	1,306 (37.7)	119 (25.2)	1,187 (39.7)	0.31	32.7	32.7	0.00
2016–2019	1,168 (33.7)	304 (64.3)	864 (28.9)	0.76	53.0	53.0	0.00
Postoperative complication within 30 days of surgery	1,022 (29.5)	143 (30.2)	879 (29.4)	0.02	29.2	29.2	0.00
Diagnosis to surgery interval in days, mean (IQR)	16.9 (0–29)	18.9 (0–33)	16.6 (0–29)	0.10	18.6	18.6	0.00
Surgery to adjuvant therapy interval in days, mean (IQR)	55.0 (40–68)	56.0 (40–70)	54.8 (40–68)	0.06	55.8	55.8	0.00
FOLFOX (versus CAPOX)	3,108 (89.8)	319 (67.4)	2,789 (93.3)	0.69	80.2	80.2	0.00
Dose reduction	1,205 (34.8)	109 (23.0)	1,096 (36.7)	0.30	27.3	27.3	0.00
Chemotherapy complication	1,127 (32.6)	147 (31.1)	980 (32.8)	0.04	29.2	29.2	0.00

Abbreviation: FRI: fall-related injury; ADG: Aggregated Diagnosis Groups. Cells containing fewer than 6 individuals and adjacent cells are suppressed to mitigate the risk of reidentification. Percentages may not sum to 100 due to suppression and rounding

In the analysis of post-treatment FRI, median follow-up was 4.6 years. Total follow-up was 17,968 person-years, during which 508 (14.7%) individuals experienced a FRI and 692 (20.0%) individuals died. The crude rate of post-treatment FRI in the cohort was 2.8 (95% CI 2.6 to 3.1) FRI per 100 person-years.

In the analysis of post-treatment fracture, median follow-up was 4.9 years. Total follow-up was 18,680 person-years, during which 301 (8.7%) individuals experienced a fracture and 733 (21.2%) individuals died. The crude rate of post-treatment fracture in the cohort was 1.6 (95% CI 1.4 to 1.8) fractures per 100 person-years). Forearm fractures were most common, followed by hip, rib, humerus, and ankle fractures (Table 2).

**Table 2:** Anatomic distribution of fractures, considering only first fractures

Fracture site	No., (%)
All	301 (100)
Forearm	92 (30.6)
Hip	79 (26.2)
Ankle	24 (8.0)
Humerus	25 (8.3)
Rib	25 (8.3)
Tibia/fibula	15 (5.0)
Other	41 (13.6)

In the overlap-weighted cohort, the distribution of weights was appropriate, all standardized differences were < 0.1, and cumulative distribution functions for continuous variables in each treatment group were qualitatively similar, indicating the PS was adequately specified.

**Fig. 2:**
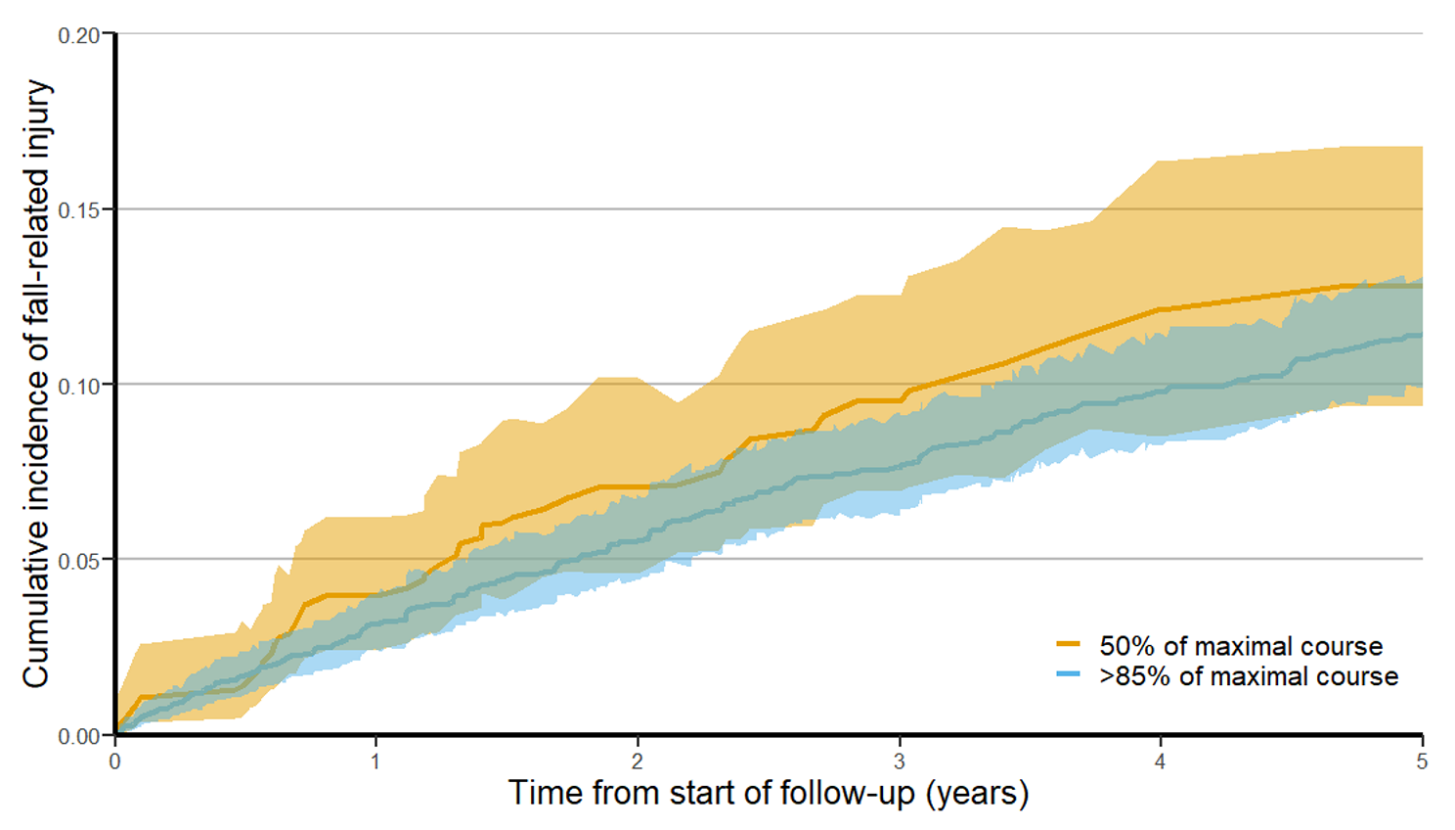
Cumulative incidence function for post-treatment fall-related injuries stratified by treatment group, with death as a competing risk and after applying overlap weights. Shaded areas represent 95% confidence intervals. Cumulative incidence functions illustrate the proportion of patients having experienced the event of interest over time, with the denominator being all patients at risk for the event at all points in time

**Fig. 3:**
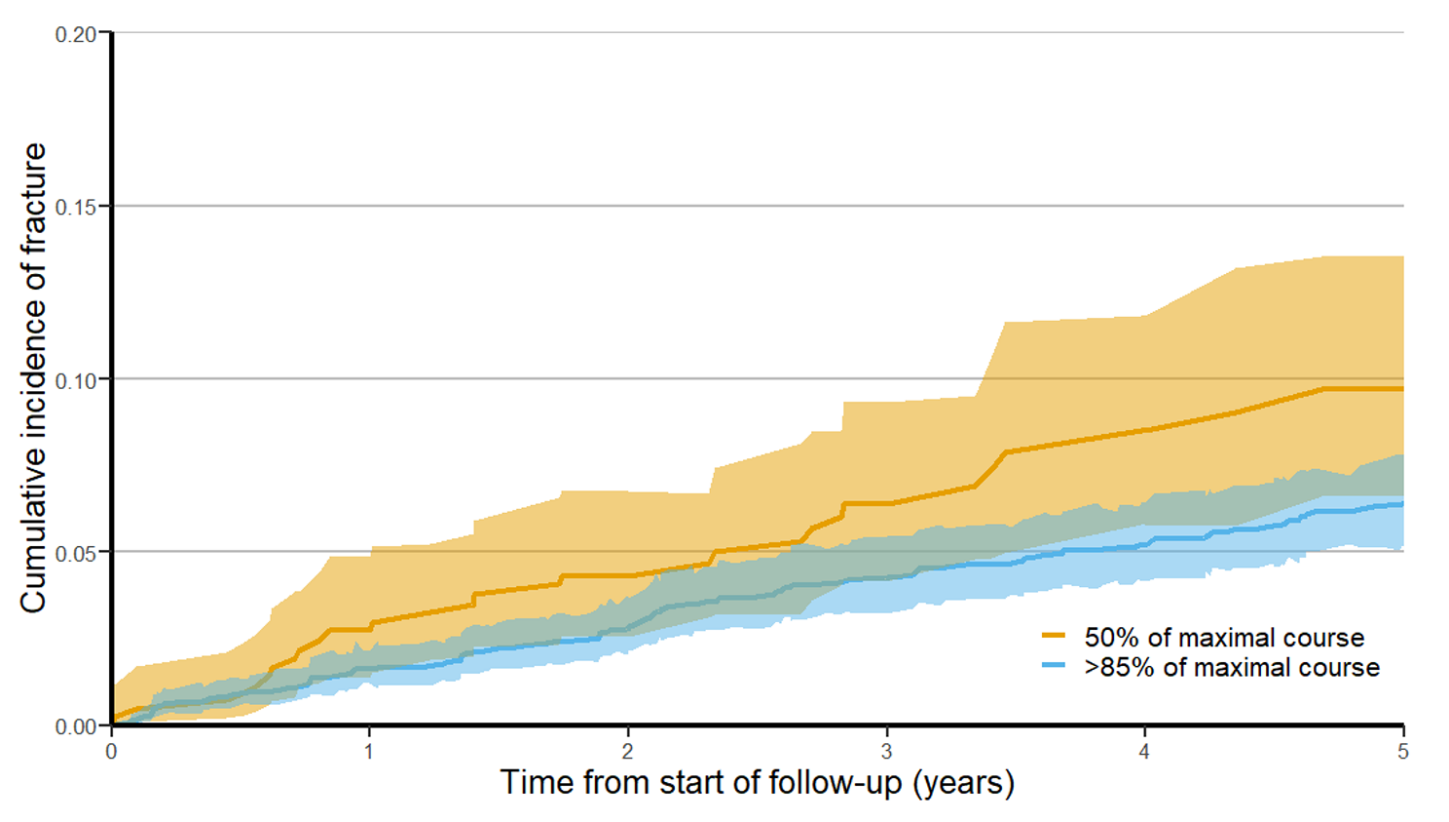
Cumulative incidence function for post-treatment fractures stratified by treatment group, with death as a competing risk and after applying overlap weights. Shaded areas represent 95% confidence intervals. Cumulative incidence functions illustrate the proportion of patients having experienced the event of interest over time, with the denominator being all patients at risk for the event at all points in time

The sHR obtained from the Fine and Gray regression model for the association of post-treatment FRI and treatment with > 85% of a maximal course of therapy was 0.84 (95% CI 0.62 to 1.13). The sHR for post-treatment fracture associated with receipt of > 85% of a maximal course of therapy was 0.72 (95% CI 0.49 to 1.06). The *p*-values for the tests of multiplicative and additive interaction for age, sex, and tumor risk were not statistically significant for either outcome. The estimated CIF for post-treatment FRI and fracture are illustrated in Figs. 2 and 3.

### Sensitivity analyses

Our estimates were similar when we compared treatment with 50% versus 100% of a maximal course of therapy, accounted for recurrent events using Andersen-Gill models, or included days between the last oxaliplatin cycle and time zero of follow-up in the overlap-weighted Fine and Gray subdistribution hazards model (data not shown).

## Discussion

Concern for elevated risk of post-treatment FRI and fracture with longer durations of oxaliplatin-containing adjuvant chemotherapy is grounded in biological plausibility, clinical experience, and limited existing research linking oxaliplatin, PN, and FRI [10, 11, 15, 17, 18, 34]. In this population-based retrospective cohort study of 3,461 patients with stage III colon cancer, post-treatment FRI and fracture were common in patients who received 50% or > 85% of a maximal course of oxaliplatin-containing adjuvant chemotherapy. However, we did not find that patients who received > 85% of a maximal course were at increased risk of post-treatment FRI or fracture compared to those who received only 50% of a maximal course, and this finding held across tumor risk, sex, and age.

Our study builds on four observational studies that examined the association between chemotherapy-associated PN and risk of self-reported falls [14–18]. Most similar to the present study is a population-based observational study using SEER-Medicare data that compared the rate of falls among cancer patients who received neurotoxic versus non-neurotoxic chemotherapy [18]. In their stratified analysis of patients with colorectal cancer, they found a hazard ratio for FRI of 1.14 comparing receipt of any neurotoxic agent against no neurotoxic agents, raising the question of whether greater extent of oxaliplatin treatment might increase the risk of FRI.

Yet, this hypothesis is not borne out in our results. Instead, our findings support the idea that the rate of FRI requiring medical care and fracture is similar within a cohort of patients with colon cancer who all received adjuvant oxaliplatin for durations relevant to the post-IDEA era. Potential mechanisms for this finding include the possibility that chemotherapy prescribers can identify those patients who would be tolerant of longer durations of chemotherapy without dramatically reducing their functional status or increasing their risk of FRI. Patients who receive more therapy may also recognize their neuropathy and take effective compensatory measures to avoid post-treatment FRI [44].

Several aspects may explain why the findings of the SEER-Medicare study differed from our observations. First, the study compared neurotoxic versus non-neurotoxic chemotherapy, rather than exploring the risk of falls within a cohort of patients who all received neurotoxic chemotherapy but of differing durations. Second, the SEER-Medicare cohort included patients with advanced cancer, potentially increasing the frailty of their population and subsequently the rate of FRI associated with neurotoxic chemotherapy. Third, our outcomes differed, where we used injuries with a fall-related mechanism and the previous study used injury codes without a specific mechanism (e.g. ‘Concussion with no loss of consciousness’).

Our study has numerous strengths. We included patients who had all received adjuvant oxaliplatin, contributing to the ongoing examination of the costs and benefits of differing durations of oxaliplatin-containing adjuvant chemotherapy for patients with stage III colon cancer. By using an overlap-weighting approach, we accounted for baseline between-group differences in important patient characteristics. Our landmark analysis mitigated immortal time bias, and the subdistribution hazards model addressed the substantial competing risk of death. Our findings were robust to multiple sensitivity analyses. Effect modifiers were thoroughly explored using tests for multiplicative and additive interaction. Finally, our study benefited from an extended duration of follow-up and the reliable capture of events resulting in hospital presentation from high-quality linked databases drawing on a population-based sample.

Several limitations of our study should be acknowledged. Our datasets capture only the most clinically significant FRI that require interaction with the healthcare system, which is not exhaustive of the total burden of post-treatment FRI [15, 45]. Partially measured and unmeasured covariates, including physical activity, visual acuity, and seizure disorder may be common causes of both a patient’s likelihood of continuing treatment and post-treatment FRI, biasing the long treatment group toward a lower rate of FRI [46]. Lastly, our data were compatible with a sHR for FRI as high as 1.13 and thus an association between FRI and longer durations of therapy cannot be definitively ruled out, despite our population-based approach and long-term follow-up. This underscores the need for future evidence syntheses of large, high-quality studies to better define this potential association.

## Conclusion

In our population-based retrospective cohort study of 3,461 patients treated with oxaliplatin-containing adjuvant chemotherapy for stage III colon cancer, treatment with > 85% of a maximal course of therapy was not associated with a significant increase the rate of post-treatment FRI or fracture over treatment with 50% of a maximal course. Though oxaliplatin is known to cause peripheral neuropathy in a dose-dependent fashion, longer durations of treatment do not appear to translate into elevated population rates of post-treatment FRI or fracture in the context of routine practice.

### Electronic supplementary material

Below is the link to the electronic supplementary material.

Supplementary Material 1

## Data Availability

The dataset from this study is held securely in coded form at ICES. While legal data sharing agreements between ICES and data providers (e.g., healthcare organizations and government) prohibit ICES from making the dataset publicly available, access may be granted to those who meet pre-specified criteria for confidential access, available at www.ices.on.ca/DAS (email: das@ices.on.ca). The full dataset creation plan and underlying analytic code are available from the authors upon request, understanding that the computer programs may rely upon coding templates or macros that are unique to ICES and are therefore either inaccessible or may require modification.
