# Supplementary material for: Association of oxaliplatin-containing adjuvant duration with post-treatment fall-related injury and fracture in patients with stage III colon cancer: a population-based retrospective cohort study
Source: BMC Cancer. 2024 Jul 22;24:878. doi: 10.1186/s12885-024-12558-2 (PMC11265086; doi:10.1186/s12885-024-12558-2)
Supplement: Supplementary file 1 — Supplementary Material 1 [file 12885_2024_12558_MOESM1_ESM.docx]

**Online-only Tables**

| **Online-only Table 1. The RECORD statement – checklist of items, extended from the STROBE statement that should be reported in observational studies using routinely collected health data.** | | | | | |  |
| --- | --- | --- | --- | --- | --- | --- |
|  | **Item No.** | **STROBE items** | **Location in manuscript where items are reported** | **RECORD items** | **Location in manuscript where items are reported** |  |
| **Title and abstract** | | | | | |  |
|  | 1 | (a) Indicate the study’s design with a commonly used term in the title or the abstract (b) Provide in the abstract an informative and balanced summary of what was done and what was found | Line 2-4, Line 86-114 | RECORD 1.1: The type of data used should be specified in the title or abstract. When possible, the name of the databases used should be included. | Line 92 |  |
|  |  |  |  |  |  |  |
|  |  |  |  | RECORD 1.2: If applicable, the geographic region and timeframe within which the study took place should be reported in the title or abstract. | Line 92-96 |  |
|  |  |  |  |  |  |  |
|  |  |  |  | RECORD 1.3: If linkage between databases was conducted for the study, this should be clearly stated in the title or abstract. | Line 92 |  |
|  |  |  |  |  |  |  |
|  |  |  |  |  |  |  |
| **Introduction** | | | | | |  |
| Background rationale | 2 | Explain the scientific background and rationale for the investigation being reported | Line 128-154 |  |  |  |
| Objectives | 3 | State specific objectives, including any prespecified hypotheses | Line 149-154 |  |  |  |
| **Methods** | | | | | |  |
| Study Design | 4 | Present key elements of study design early in the paper | Line 157-158 |  |  |  |
| Setting | 5 | Describe the setting, locations, and relevant dates, including periods of recruitment, exposure, follow-up, and data collection | Line 166-237 |  |  |  |
| Participants | 6 | *(a) Cohort study* - Give the eligibility criteria, and the sources and methods of selection of participants. Describe methods of follow-up | Line 166-237 | RECORD 6.1: The methods of study population selection (such as codes or algorithms used to identify subjects) should be listed in detail. If this is not possible, an explanation should be provided. | Line 166-237, Online-only Table 2 |  |
|  |  | *Case-control study* - Give the eligibility criteria, and the sources and methods of case ascertainment and control selection. Give the rationale for the choice of cases and controls |  |  |  |  |
|  |  | *Cross-sectional study* - Give the eligibility criteria, and the sources and methods of selection of participants |  | RECORD 6.2: Any validation studies of the codes or algorithms used to select the population should be referenced. If validation was conducted for this study and not published elsewhere, detailed methods and results should be provided. | Line 166-237, Online-only Table 2 |  |
|  |  | *(b) Cohort study* - For matched studies, give matching criteria and number of exposed and unexposed |  | RECORD 6.3: If the study involved linkage of databases, consider use of a flow diagram or other graphical display to demonstrate the data linkage process, including the number of individuals with linked data at each stage. |  |  |
|  |  | *Case-control study* - For matched studies, give matching criteria and the number of controls per case |  |  |  |  |
| Variables | 7 | Clearly define all outcomes, exposures, predictors, potential confounders, and effect modifiers. Give diagnostic criteria, if applicable. | Line 166-237 | RECORD 7.1: A complete list of codes and algorithms used to classify exposures, outcomes, confounders, and effect modifiers should be provided. If these cannot be reported, an explanation should be provided. | Online-only Table 2 |  |
| Data sources/ measurement | 8 | For each variable of interest, give sources of data and details of methods of assessment (measurement). | Line 166-237, Online-only Table 2 |  |  |  |
|  |  | Describe comparability of assessment methods if there is more than one group |  |  |  |  |
| Bias | 9 | Describe any efforts to address potential sources of bias | Line 244-279 |  |  |  |
| Study size | 10 | Explain how the study size was arrived at | Figure 1 |  |  |  |
| Quantitative variables | 11 | Explain how quantitative variables were handled in the analyses. If applicable, describe which groupings were chosen, and why | Line 166-237 |  |  |  |
| Statistical methods | 12 | (a) Describe all statistical methods, including those used to control for confounding (b) Describe any methods used to examine subgroups and interactions (c) Explain how missing data were addressed (d) *Cohort study* - If applicable, explain how loss to follow-up was addressed *Case-control study* - If applicable, explain how matching of cases and controls was addressed *Cross-sectional study* - If applicable, describe analytical methods taking account of sampling strategy (e) Describe any sensitivity analyses | Line 239-271 |  |  |  |
| Data access and cleaning methods |  |  |  | RECORD 12.1: Authors should describe the extent to which the investigators had access to the database population used to create the study population. | Line 159-165 |  |
|  |  |  |  | RECORD 12.2: Authors should provide information on the data cleaning methods used in the study. |  |  |
| Linkage |  |  |  | RECORD 12.3: State whether the study included person-level, institutional-level, or other data linkage across two or more databases. The methods of linkage and methods of linkage quality evaluation should be provided. | Line 159-165 |  |
| **Results** | | | | | |  |
| Participants | 13 | (a) Report the numbers of individuals at each stage of the study (*e.g.*, numbers potentially eligible, examined for eligibility, confirmed eligible, included in the study, completing follow-up, and analysed) |  | RECORD 13.1: Describe in detail the selection of the persons included in the study (*i.e.,* study population selection) including filtering based on data quality, data availability and linkage. The selection of included persons can be described in the text and/or by means of the study flow diagram. | Figure 1 |  |
|  |  | (b) Give reasons for non-participation at each stage. |  |  |  |  |
|  |  | (c) Consider use of a flow diagram |  |  |  |  |
| Descriptive data | 14 | (a) Give characteristics of study participants (*e.g.*, demographic, clinical, social) and information on exposures and potential confounders | Table 1, Line 281-295 |  |  |  |
|  |  | (b) Indicate the number of participants with missing data for each variable of interest |  |  |  |  |
|  |  | (c) *Cohort study* - summarise follow-up time (*e.g.*, average and total amount) |  |  |  |  |
| Outcome data | 15 | *Cohort study* - Report numbers of outcome events or summary measures over time | Line 281-295 |  |  |  |
|  |  | *Case-control study* - Report numbers in each exposure category, or summary measures of exposure |  |  |  |  |
|  |  | *Cross-sectional study* - Report numbers of outcome events or summary measures |  |  |  |  |
| Main results | 16 | (a) Give unadjusted estimates and, if applicable, confounder-adjusted estimates and their precision (e.g., 95 confidence interval). Make clear which confounders were adjusted for and why they were included | Line 299-304, Figure 2, Figure 3 |  |  |  |
|  |  | (b) Report category boundaries when continuous variables were categorized |  |  |  |  |
|  |  | (c) If relevant, consider translating estimates of relative risk into absolute risk for a meaningful time period |  |  |  |  |
| Other analyses | 17 | Report other analyses done—e.g., analyses of subgroups and interactions, and sensitivity analyses | Line 306-309 |  |  |  |
| **Discussion** | | | | | |  |
| Key results | 18 | Summarise key results with reference to study objectives | Line 311-319 |  |  |  |
| Limitations | 19 | Discuss limitations of the study, taking into account sources of potential bias or imprecision. Discuss both direction and magnitude of any potential bias | Line 341-357 | RECORD 19.1: Discuss the implications of using data that were not created or collected to answer the specific research question(s). Include discussion of misclassification bias, unmeasured confounding, missing data, and changing eligibility over time, as they pertain to the study being reported. | Line 350-357 |  |
| Interpretation | 20 | Give a cautious overall interpretation of results considering objectives, limitations, multiplicity of analyses, results from similar studies, and other relevant evidence | Line 370-376 |  |  |  |
| Generalisability | 21 | Discuss the generalisability (external validity) of the study results | Line 365-368 |  |  |  |
| **Other Information** | | | | | |  |
| Funding | 22 | Give the source of funding and the role of the funders for the present study and, if applicable, for the original study on which the present article is based | Line 37-45 |  |  |  |
| Accessibility of protocol, raw data, and programming code |  | .. |  | RECORD 22.1: Authors should provide information on how to access any supplemental information such as the study protocol, raw data, or programming code. | Online-only Appendix |  |

| **Online-only Table 2. Exposure, outcome, and patient characteristics with coding algorithms.** | | |
| --- | --- | --- |
| **Characteristic** | **Data source** | **Codes** |
| Sex | RPDB | RPDB SEX=M or F |
| Colon cancer diagnosis | OCR | Proximal colon: ICD-O-3 Topography code C180, C182-C184 Distal colon: ICD-O-3 Topography code C185-C187, C199^1^ |
| Colon resection | DAD | CCI 1NM76, 1NM77, 1NM87, 1NM89, 1NM91, 1NQ87, 1NQ89^1–3^ |
| Chemotherapy within 5 years prior to colon cancer diagnosis | OHIP, NACRS, NDFP, ALR | OHIP FEECODE: G281, G339, G345, G359, G381, G382, G388, K070 (medical oncologist billing for CCAC infusion) NACRS DX10CODE: Z5211, Z512 NDFP: any record with a valid drug name ALR: any record with a valid CCO regimen |
| ADG Score^4^ | NACRS, DAD, OHIP | ADG comorbidity score derived from weighted ADG categories present during 2-year lookback at outpatient and inpatient records |
| Frailty | NACRS, DAD, OHIP | ACG Flag FRAILTY = yes |
| Diabetes | DAD, OHIP, ODB | ICES derived cohort  2 OHIP dxcode 250 claims or 1 ODB DM drug claim or 1 DAD admission within 1 year |
| Dementia | DAD, NACRS, OHIP, ODB | ICES derived cohort  The person had at least 3 OHIP claims with a dementia diagnosis recorded which were each at least 30 days apart in a 2-year period; or the person had at least one hospitalization or same day surgery with a dementia diagnosis recorded; or the person had at least one ODB claim with a dementia medication dispensed |
| Osteoporosis^5^ | NACRS, DAD, OHIP | OHIP 733; ICD10 M80, M81, or M82 in lookback window or follow-up |
| Stroke^6,7^ | NACRS, DAD | ICD10 H34.1, I60.x, I61.x I63.x, I64.x69,70 |
| Alcohol-related hospital visit^8,9^ | NACRS, DAD | ICD10 F10 |
| Neuropathy^10^ | NACRS, DAD | DAD ICD10 primary diagnosis: R296, G632, G560, G629, R2688, G990, R2682, R208, R270, R262, R298, G628, G562, R202, G590, R278, G588, G130, G571, G620, G572, G610, G633, G563, G568, G569, G576, G578, G579, G589, G608, G618, G622, G630, G631, G635, G636, G900, M7925, M7928, R201, R203  NACRS ICD10 primary diagnosis: G631, G620, G629, R296, G632, R208, R202, R2682, R2688, R270, G560, R298, G628, M7928, G579, R262, G990, G590, G562, G622, G630, R278, G571, G589, G900, M7925, M7926, M7929, G561, G598, G618, G636, M7927, R203 |
| Deprivation quintile | CENSUS | GETONMARG macro variable deprivation_q_da, based on most recent dissemination area prior to first adjuvant treatment date |
| Rurality | CENSUS | binary variable rural = 1 if PCCF rural flag = Y |
| Oxaliplatin | NDFP | DRUG_NAME = 'Oxaliplatin' |
| Adjuvant regimen | NDFP, ALR, OHIP, ODB | Hierarchical algorithm criteria:  1. Modal cycle interval by NDFP: if 18-24 days between cycles, then CAPOX; if 11-17 days between cycles then FOLFOX 2. First cycle interval by NDFP: if 18-24 days between cycles, then CAPOX; if 11-17 days between cycles then FOLFOX 3. Modal oxaliplatin-containing regimen by ALR 4. First oxaliplatin-containing regimen by ALR 5. If received oxaliplatin and OHIP billing code G388 for oral chemotherapy during exposure window, then CAPOX 6. If received oxaliplatin and ODB claim for oral chemotherapy (DIN 02426765, 02457504, 02421917, 02457490, 02426757, 02400022, 02238453, 02421925, 02400030, 02238454) during exposure window, then CAPOX |
| Postoperative complication within 30 days of index operation ^11^ | DAD, NACRS, OHIP | Reoperation for intra-abdominal complication  CCI |
|  |  | 1.NK.80.^^,1.NM.52.^^,1.NM.80.^^,1.NP.86.^^,1.OT.13.^^,1.OT.52.^^,1.OT.70.LA,1.NK.76.^^,1.NK.77.^^,1.NK.87.^^,1.NM.76.^^,1.NM.77.^^,1.NM.87.^^,1.NM.89.^^,1.NM.91.^^ |
|  |  |  |
|  |  | Venous thromboembolism or pulmonary embolism |
|  |  | ICD10 |
|  |  | I.26.^^,I.80.1-I.80.3 |
|  |  |  |
|  |  | Sepsis |
|  |  | ICD10 |
|  |  | A.41.^^,A.41.1.,A.41.2.,A.41.3.,A.41.4.,A.41.5.^^,A.41.8.^^,A.41.9. |
|  |  |  |
|  |  | Hemorrhage |
|  |  | CCI |
|  |  | 1.LZ.19^^ |
|  |  | ICD10 |
|  |  | T.81.0,T81.1,R.58. |
|  |  |  |
|  |  | Percutaneous drainage of abdominal abscess |
|  |  | OHIP |
|  |  | S313,S314,Z569,Z594 |
|  |  |  |
|  |  | Major wound disruption |
|  |  | CCI |
|  |  | 1.SY.80^^ |
|  |  | ICD10 |
|  |  | T.81.3. |
|  |  | OHIP |
|  |  | S343 |
|  |  |  |
|  |  | Fistula formation |
|  |  | CCI |
|  |  | 1.NP.86.^^ |
|  |  | ICD10 |
|  |  | K.63.2.,K.31.6.,N.32.1. |
|  |  | OHIP |
|  |  | E714 |
|  |  |  |
|  |  | Wound infection |
|  |  | ICD10 |
|  |  | T.81.4. |
|  |  |  |
|  |  | Stroke or transient ischemic attack |
|  |  | ICD10 G.45.^^,I.60.^^,I.61.^^,I.63.^^,I.64.,H.34.1 |
|  |  |  |
|  |  | Myocardial infarction |
|  |  | ICD10 I.21.^^,I.22.^^,I.23.^^ |
|  |  |  |
|  |  | Congestive Heart Failure |
|  |  | ICD10 I.50.^^ |
| Dose reduction ^12^ | NDFP | Any oxaliplatin dose <80 of the first dose |
| Chemotherapy complication requiring ED visit or hospital admission^13–16^ | DAD, NACRS | ICD10 in any diagnostic space |
|  |  | Neutropenia |
|  |  | Agranulocytosis (D70.*) |
|  |  | Fever |
|  |  | Fever of unknown origin (R50.*) |
|  |  | Infection |
|  |  | Infectious and parasitic disease, including sepsis (A00.*-B99.*) |
|  |  | Infection and inflammatory reaction due to other cardiac and vascular devices, implants and grafts (T82.7) |
|  |  | Bronchitis (J20.*-J22.*) |
|  |  | Pneumonia (J09.*-J11.*) |
|  |  | Kidney infection (N10, N39.0) |
|  |  | Acute cystitis (N30.0) |
|  |  | Cellulitis (L00.*-L08.*) |
|  |  | Empyema (J86.*) |
|  |  | Abscess lung/mediastinum (J85.*) |
|  |  | GI Toxicity |
|  |  | Diarrhea, colitis (K52.*) |
|  |  | Functional diarrhea (K59.1) |
|  |  | Nausea and vomiting (R11.*) |
|  |  | Heartburn (R12.*) |
|  |  | Constipation (K59.0) |
|  |  | Obstruction (includes ileus) (K56.*) |
|  |  | Stomatitis (K12.*) |
|  |  | Cachexia (R64.*) |
|  |  | Anorexia (R63.0) |
|  |  | Other systemic treatment related |
|  |  | Hyponatremia (E87.1) |
|  |  | Hypokalemia (E87.6) |
|  |  | Other electrolyte/fluid abnormality (E87.*) |
|  |  | Magnesium disorder (E83.4) |
|  |  | Dehydration/hypovolemia (E86.*) |
|  |  | Malaise/fatigue (R53.*) |
|  |  | Syncope (R55.*) |
|  |  | Dizziness (R42.*) |
|  |  | Hypotension (I95.9) |
|  |  | Fe deficiency anemia (D50.*) |
|  |  | Other deficiency anemia (D51.*-D53.*) |
|  |  | Aplastic anemia (D60.*-D61.*) |
|  |  | Other and unspecified anemia (D62.*-D64.*) |
|  |  | Thrombocytopenia (D69.5, D69.6) |
|  |  | Other venous embolism and thrombosis (I82.*) |
|  |  | Rash and non-specific skin eruptions (R21.*) |
|  |  | Hyperglycemia (R73.*) |
|  |  | Phlebitis and thrombophlebitis (I80.*) |
|  |  | Pulmonary embolism (I26.*) |
|  |  | Disorders of calcium metabolism (E83.5) |
|  |  | Disorders of phosphorus metabolism and phosphatases (E83.3) |
| Fall-related injury* | NACRS, DAD | ICD 10 W00-W19 |
| Fractureⱡ^17–21^ | NACRS, DAD, OHIP | Hip/Femur (ICD-10 S720, S721, S723; CCI 1VA73, 1VC73, 1VA74, 1VA53, 1VC74, 1VA80, 1VC03, 1VC80; OHIP F095, F096, F097, Z211) |
|  |  | Forearm (ICD 10 S52; CCI 1TV73, 1TV74, 1TV03; OHIP F014, F022, F023, F025, F026, F028, F030, F032, F033, F046, F024, F027, F031, Z203) |
|  |  | Humerus (ICD-10 S422, S423, S424) |
|  |  | Vertebral (ICD-10 S220, S221, S320, T080, T081) |
|  |  | Pelvis (ICD-10 S321, S322, S324, S323, S325, S327, S328) |
|  |  | Ankle (ICD-10 S825, S826, S827, S828, S829) |
|  |  | Patella (ICD-10 S820) |
|  |  | Tibia/Fibula (ICD-10 S821, S822, S823, S824) Ribs/sternum (ICD 10 S222, S223, S224) |
|  |  | Trunk (ICD-10 S229) |
|  |  | Scapula (ICD-10 S421) |
|  |  | Clavicle (ICD-10 S420) |
| Abbreviation: ADG, Aggregated Diagnosis Group; AJCC, American Joint Committee on Cancer; ALR, Activity Level Registry; CCI, Canadian Classification of Interventions; DAD, Discharge Abstract Database; NACRS, National Ambulatory Care Reporting System; NDFP, New Drug Funding Program; OCR, Ontario Cancer Registry; OHIP, Ontario Health Insurance Plan; RPDB, Registered Persons' Database  *The positive predictive value for ICD-10 fall-related injury codes is 0.91 (0.86 to 0.94) against medical chart abstraction for fall-related presentations to hospital (personal communication, Blayne Welk, September 2021).  ⱡThe performance characteristics of the codes for fracture depend on the fracture site. ICD10 S72 for femur fracture: K 0.95 (0.94 to 0.97), sensitivity 0.95 (0.93 to 0.97), PPV 0.95 (0.92 to 0.97).^20^ CCI 1VC74 for femur fixation: K 0.86 (0.82 to 0.90) sensitivity 0.89 (0.83 to 0.93) PPV 0.83 (0.77 to 0.88). Orthopedic surgeon billing codes for hip/femur fracture: PPV 0.83 (0.79 to 0.87)^18^ | | |

1. Tan J. *THE PROCESSES OF CARE AFTER COLORECTAL CANCER SURGERY IN ONTARIO*.; 2008. Accessed June 15, 2020. https://tspace.library.utoronto.ca/handle/1807/17228

2. Paszat LF, Sutradhar R, Corn E, et al. Morbidity and mortality following major large bowel resection for colorectal cancer detected by a population-based screening program. *J Med Screen*. Published online 2020. doi:10.1177/0969141320957361

3. Booth CM, Nanji S, Wei X, et al. Adjuvant Chemotherapy for Stage II Colon Cancer: Practice Patterns and Effectiveness in the General Population. *Clin Oncol*. 2017;29(1):e29-e38. doi:10.1016/j.clon.2016.09.001

4. Austin PC, Walraven C van. The mortality risk score and the ADG score: Two points-based scoring systems for the Johns Hopkins aggregated diagnosis groups to predict mortality in a general adult population Cohort in Ontario, Canada. *Med Care*. 2011;49(10):940-947. doi:10.1097/MLR.0b013e318229360e

5. Cadarette SM, Jaglal SB, Raman-Wilms L, Beaton DE, Paterson JM. Osteoporosis quality indicators using healthcare utilization data. *Osteoporosis International*. 2011;22(5):1335-1342. doi:10.1007/s00198-010-1329-8

6. Hall R, Mondor L, Porter J, Fang J, Kapral MK. Accuracy of administrative data for the coding of acute stroke and TIAs. *Canadian Journal of Neurological Sciences*. 2016;43(6):765-773. doi:10.1017/cjn.2016.278

7. Kokotailo RA, Hill MD. Coding of stroke and stroke risk factors using International Classification of Diseases, revisions 9 and 10. *Stroke*. 2005;36(8):1776-1781. doi:10.1161/01.STR.0000174293.17959.a1

8. Kool B, Ameratunga S, Jackson R. The role of alcohol in unintentional falls among young and middle-aged adults: A systematic review of epidemiological studies. *Injury Prevention*. 2009;15(5):341-347. doi:10.1136/ip.2008.021303

9. Hulme J, Sheikh H, Xie E, Gatov E, Nagamuthu C, Kurdyak P. Mortality among patients with frequent emergency department use for alcohol-related reasons in Ontario: a population-based cohort study. *CMAJ*. 2020;192(47):1522-1531. doi:10.1503/cmaj.191730

10. Raphael MJ, Fischer HD, Fung K, et al. Neurotoxicity Outcomes in a Population-based Cohort of Elderly Patients Treated With Adjuvant Oxaliplatin for Colorectal Cancer. *Clin Colorectal Cancer*. 2017;16(4):397-404.e1. doi:10.1016/j.clcc.2017.03.013

11. Baxter NN, Fischer HD, Richardson DP, et al. A population-based study of complications after colorectal surgery in patients who have received bevacizumab. *Dis Colon Rectum*. 2018;61(3):306-313. doi:10.1097/DCR.0000000000000966

12. Karim S, Wei X, Leveridge MJ, et al. Delivery of chemotherapy for testicular cancer in routine practice: A population-based study. *Urologic Oncology: Seminars and Original Investigations*. 2019;37(3):183.e17-183.e24. doi:10.1016/j.urolonc.2018.10.025

13. Grewal K, Sutradhar R, Krzyzanowska MK, Redelmeier DA, Atzema CL. The association of continuity of care and cancer centre affiliation with outcomes among patients with cancer who require emergency department care. *CMAJ*. 2019;191(16):E436-E445. doi:10.1503/cmaj.180962

14. Krzyzanowska MK, Enright K, Moineddin R, et al. Can chemotherapy-related acute care visits be accurately identified in administrative data? *J Oncol Pract*. 2018;14(1):e51-e58. doi:10.1200/JOP.2017.023697

15. Cancer and Leukemia Group B. *CALGB/SWOG 80702: A Phase III Trial of 6 versus 12 Treatments of Adjuvant FOLFOX plus Celecoxib or Placebo for Patients with Resected Stage III Colon Cancer: Trial Protocol*.; 2010. doi:10.1158/1078-0432.CCR-06-9010

16. Boyne DJ, Cheung WY, Hilsden RJ, et al. Association of a Shortened Duration of Adjuvant Chemotherapy with Overall Survival among Individuals with Stage III Colon Cancer. *JAMA Netw Open*. 2021;4(3):1-11. doi:10.1001/jamanetworkopen.2021.3587

17. Welk B, McArther E, Fraser LA, et al. The risk of fall and fracture with the initiation of a prostate-selective α antagonist: A population based cohort study. *Br Med J*. 2015;351(h5398). doi:10.1016/j.juro.2016.02.040

18. Jean S, Candas B, Belzile É, et al. Algorithms can be used to identify fragility fracture cases in physician-claims databases. *Osteoporosis International*. 2012;23(2):483-501. doi:10.1007/s00198-011-1559-4

19. Naylor KL, Jamal SA, Zou G, et al. Fracture incidence in adult kidney transplant recipients. *Transplantation*. 2016;100(1):167-175. doi:10.1097/TP.0000000000000808

20. Juurlink D, Preyra C, Croxford R, et al. *Canadian Institute for Health Information Discharge Abstract Database: A Validation Study*.; 2006. https://www.ices.on.ca/~/media/Files/Atlases-Reports/2006/CIHI-DAD-a-validation-study/Full-report.ashx

21. Stone CA, Lawlor PG, Savva GM, Bennett K, Kenny RA. Prospective study of falls and risk factors for falls in adults with advanced cancer. *Journal of Clinical Oncology*. 2012;30(17):2128-2133. doi:10.1200/JCO.2011.40.7791
